# The consequences of ISO 45001: preliminary analysis of cases in Korea

**DOI:** 10.1093/joccuh/uiad007

**Published:** 2023-11-14

**Authors:** Ikhyun Joo, Kyungmin Baek

**Affiliations:** Institute of Defense Acquisition Program, Kwangwoon University, Seoul 01897, Korea; Information Sociology Department, Soongsil University, Seoul 06978, Korea

**Keywords:** ISO 45001, occupational safety and health, management standard

## Abstract

**Objectives:** Using cases in the Korean workplace, this study preliminarily investigated whether ISO 45001-certified and non-certified workplaces differ in 4 aspects of occupational safety and health (OSH) management.

**Methods:** Data were obtained from the 2021 Survey on the Status of Safety and Health in the Workplace in Korea. This study used a matched-pair analysis of certified and non-certified workplaces.

**Results:** The results suggest that although ISO 45001-certified workplaces have a more safety-friendly internal institutional context than non-certified workplaces, there is no significant difference in the number of injuries and fatalities.

**Conclusions:** The results indicate that ISO 45001-certified workplaces do not achieve better OSH performance than non-certified workplaces because ISO 45001 remains in the early stages of expansion, and certification does not require the achievement of OSH performance. A longitudinal analysis is needed to confirm the accurate outcomes of ISO 45001 certification.

## Teaser

**Key points:**

- **What is already known on this topic**: Business organizations worldwide have become certified as ISO 45001 to ensure the safety and health of workers.
- **What this study adds**: This study contributes to understanding the consequences of ISO 45001, broadly known as the OSH standard.
- **How this study might affect research, practice, or policy**: This study provides preliminary results to expand understanding of ISO 45001, broadly known as the OSH standard.

## 1. Introduction

Workplace safety and health has recently been in the spotlight, with industrial accidents garnering increasing attention. As occupational safety and health (OSH) acts worldwide have become more stringent, OSH has become an important consideration in business operations. Unsafe and unhealthy operations in workplaces are not only penalized by regulators and criticized by the public but also affect the reputation and performance of the corresponding firms.^1^ Industrial accidents can lead to financial losses for companies, not just human losses. Growing public attention to OSH has led to a shift in business thinking that safety and health are not costs but essential business investments, and this has recently become an important motivation for businesses to achieve OSH-oriented management standards.

Among the various management standards, ISO 45001 as a sister standard of ISO 9001 and ISO 14001 was established in February 2018. With the growing societal focus on OSH, ISO 45001 has rapidly spread worldwide. As of 2021, more than 370 000 workplaces were certified as ISO 45001.^2^ However, there is a lack of research on the consequences of certification. Using cases from a Korean workplace, this study preliminarily investigated whether ISO 45001-certified and non-certified workplaces differ in 4 aspects of OSH.

As noted above, many business organizations worldwide have become certified as ISO 45001 to ensure the safety and health of workers. The main role of the ISO 45001 standard is to serve as a useful instrument to proactively improve OSH performance, regardless of the size, type, and nature of the organization.^2^ The International Organization for Standardization (ISO) developed ISO 45001 to help ISO 45001-certified organizations improve their OSH performance by integrating OSH into their business management systems and processes. Moreover, ISO 45001 emphasizes top management’s leadership and involvement in OSH management and workers’ participation in safety management, and requires that the needs of various stakeholders, including workers, be reflected in the organization’s operations. Similar to other sister ISO management standards, ISO 45001 is based on the Plan-Do-Check-Act approach (ie, procedural conformity rather than substantial changes) and does not have performance requirements.^3^

Thus, it is possible to infer the difference between ISO 45001-certified and non-certified workplaces in terms of (1) the level of integration of OSH into the business management system and its operation, (2) top management’s leadership and involvement in OSH management, (3) workers’ participation in OSH management, and (4) the number of injuries and fatalities that occurred during work. Accordingly, the following hypotheses are proposed:

Hypothesis 1: ISO 45001-certified and non-certified facilities differ in the level of integration of OSH into business management system and its operation.

Hypothesis 2: ISO 45001-certified and non-certified facilities differ in the level of top management’s leadership and involvement in OSH management.

Hypothesis 3: ISO 45001-certified and non-certified facilities differ in the level of workers’ participation in OSH management.

Hypothesis 4: ISO 45001-certified and non-certified facilities differ in the number of injuries and fatalities that occurred during the course of work.

The first 3 of the 4 hypotheses aimed to investigate the disparities between ISO 45001-certified and non-certified workplaces concerning the organizational context related to workplace safety and health. The final hypothesis aimed to explore the distinctions between ISO 45001-certified and non-certified workplaces in terms of actual workplace safety and health performance.

## 2. Methods

To determine whether ISO 45001-certified and non-certified facilities differ in various aspects of workplace safety and health, we used an analytical framework developed by Gomez and Rodriguez,^4^ Bansal and Hunter,^5^ and Baek^6^ to explore OSH performance across workplaces in Korea. Only few studies have investigated the consequences of ISO 45001 because the data are not available. Our dataset surveyed multiple questions to measure the impact of ISO 45001 on 4 aspects. However, our data were collected only once, and we were unable to conduct a longitudinal analysis. Therefore, we borrowed a research strategy using matched samples from these studies and applied it to our dataset. A matched-pair analysis is frequently used to collect and match samples with similar characteristics. The objective of this study was to investigate whether there is a statistically significant difference in the mean values of 5 dependent variables between ISO 45001-certified and non-certified workplaces. To ascertain the true impact of ISO 45001, it becomes imperative to control for workplace characteristics, such as size, industry, the presence of a dedicated occupational health and safety department, and Korea Occupational Safety and Health Agency (KOSHA) 18001 certification, on the dependent variables. Although workplace characteristics can be included as control variables in Ordinary Least Square (OLS) regression analysis, it is essential to consider that the characteristics of ISO 45001-certified and non-certified workplaces may exhibit heterogeneity. Consequently, this study employed a method known as “matching” to assess the effect of ISO 45001 on the dependent variables only within cases with homogeneous workplace characteristics. This method allowed us to conduct experimental research by examining whether there was a statistically significant difference between ISO 45100-certified and non-certified workplaces, controlling for other variables.^7^

We compared the level of OSH performance in 2 independent groups—ISO 45001-certified facilities and non-certified facilities—to determine whether there was statistical evidence that the associated populations were significantly different in terms of (1) the level of integration of OSH into the business management system and its operation, (2) top management’s leadership involvement in OSH management, (3) workers’ participation in OSH management, and (4) the number of injuries and fatalities that occurred during the course of work. To test these hypotheses, we used data from the 2021 Survey on the Status of Safety and Health in the Workplace in Korea.

### 2.1. Data

This survey was conducted in 2021 by the KOSHA, a government-sponsored research institute, to identify the status of safety and health in Korean workplaces, provide empirical data to establish OSH policies, determine priorities for future OSH projects, and prepare mid- to long-term policies.^8^ The survey was conducted by Gallup Korea in the second half of 2021 by sending surveyors to workplaces to interview safety and health representatives, who then completed a structured questionnaire based on their responses.

The survey was conducted nationwide across workplaces in the manufacturing, construction, and service sectors on 20 or more full-time workers to understand the actual situation and awareness of OSH. In workplaces with 20 or fewer employees, it was not easy to investigate safety and health activities because of the rapid creation and destruction of workplaces and the lack of safety and health management personnel, which may reduce the reliability of the survey data.

To obtain the sample, the business register of the National Business Survey of Statistics Korea was used as the sampling framework. The survey was conducted in 6000 workplaces, including 3000 in the manufacturing sector, 1500 in the construction sector, and 2500 in the service sector. This study focused only on workplaces in the manufacturing sector because industrial accidents are on the rise in the manufacturing sector.^9^

### 2.2. Variables

#### 2.2.1. The level of integration of OSH into business management

The level of integration of OSH into business management is the average of responses to 10 statements about OSH-related activities within a workplace: (1) I have ample opportunities to talk about occupational safety issues in meetings; (2) I talk openly about occupational safety issues with my colleagues; (3) My workplace regularly asks employees for their opinions on OSH; (4) My workplace has a system where I can make suggestions about occupational safety; (5) My workplace is responsive to employee suggestions about occupational safety; (6) My workplace provides ample opportunities for OSH education and training for its employees; (7) Education and training programs for OSH in my workplace reduce disasters; (8) My workplace has systematic occupational safety policies and procedures; (9) My workplace has provided its employees with the facilities, equipment, and protective clothing for occupational safely; (10) My workplace is very organized. These statements were answered on a 5-point Likert scale ranging from 1 (not at all) to 5 (absolutely). Cronbach’s α for this index was .92.

#### 2.2.2. The level of top management’s leadership and involvement in OSH management

The level of top management’s leadership and involvement in safety management is the average of responses to 3 statements about OSH-related activities within a workplace: (1) My company’s management places a strong emphasis on the OSH of workers, (2) My company’s leadership prioritizes OSH, and (3) My company’s leadership takes occupational safety seriously. These statements were answered on a 5-point Likert scale ranging from 1 (not at all) to 5 (absolutely). Cronbach’s α for this index was .88.

#### 2.2.3. The level of workers’ participation in OSH management

The level of workers’ participation in OSH management is the average of responses to 3 statements about OSH-related activities within a workplace: (1) Employees in my workplace always follow OSH procedures and work standards, (2) Employees in my workplace can refuse to work if they feel unsafe, and (3) Employees in my workplace voluntarily work to improve OSH. These statements were answered on a 5-point Likert scale ranging from 1 (not at all) to 5 (absolutely). Cronbach’s α for this index was .82.

#### 2.2.4. The number of injuries and fatalities

To estimate the number of injuries, we used the average number of employees who reported workplace accidents or work-related illnesses between 2020 and 2021. To estimate the number of fatalities, we used the average number of employees killed in workplace accidents or work-related illnesses between 2020 and 2021. The definitions of workplace accidents and work-related illnesses used in this study correspond to Article 37 of the Korean Workers’ Compensation Insurance Act.

**Table 1:** Sample descriptive statistics.

		**ISO 45001**			
		**Yes**		**No**	
		**Freq. (mean)**	**Percent (SD)**	**Freq. (mean)**	**Percent (SD)**
**Number of employees**	20-49	407	62.4	407	62.4
	50-99	126	19.3	126	19.3
	100-299	90	13.8	90	13.8
	300-499	19	2.9	19	2.9
	500-999	8	1.2	8	1.2
	1000+	2	0.3	2	0.3
**Responsible department**	Yes	94	14.4	94	14.4
	No	558	85.6	558	85.6
**KOSHA 18001 (Korean government-sponsored standard that addresses the requirements of OSHA 18001)**	Yes	49	7.5	49	7.5
	No	603	92.5	603	92.5
**The level of integration of OSH into business management**		(4.596)	(0.021)	(4.517)	(0.024)
**The level of top management’s leadership and involvement in OSH management**		(4.364)	(0.021)	(4.184)	(0.023)
**The level of workers’ participation in OSH management**		(4.315)	(0.024)	(4.155)	(0.027)
**The number of injuries**		(0.238)	(0.028)	(0.326)	(0.053)
**The number of fatalities**		(0.026)	(0.023)	(0.003)	(0.002)

Abbreviations: KOSHA, Korea Occupational Safety and Health Agency; OSH, occupational safety and health; OSHA,

**Table 2:** Results of Wilcoxson signed-rank test.

**Variable**	**The number of pairs**	***z* value**	**Significance: *P* value**
**The level of integration of OSH into business management**	652	−7.037	<.001
**The level of top management’s leadership and involvement in OSH management**	652	−2.519	<.05
**The level of workers’ participation in OSH management**	652	−4.319	<.001
**The number of injuries**	652	0.468	Not significant
**The number of fatalities**	652	−0.381	Not significant

### 2.3. Analytical strategy

Before comparing ISO 45001-certified and non-certified workplaces, it was necessary to control for other factors affecting the 4 aspects of OSH in the workplace. To do so, we used a matched-pair analysis of certified and non-certified workplaces, controlling for the pairs for workplace size based on the number of employees, OSH department, and whether a workplace was certified according to the KOSHA 18001 standard. KOSHA 18001 is a Korean government-sponsored standard that addresses the requirements of OSHA 18001. Certification to ISO 45001 could be a low-hanging fruit for organizations certified to KOSHA 18001. Therefore, we controlled for the presence of KOSHA 18001 to verify the net effect of ISO 45001. We particularly controlled for whether a workplace was certified according to the KOSHA 18001 standard because certification to ISO 45001 is a relatively low-hanging fruit for KOSHA 18001-certified workplaces, and KOSHA 18001-certified workplaces may have better OSH performance than non-certified workplaces.

First, we checked whether the distribution of differences in pairs from the matched samples came from normally distributed populations before performing a mean comparison analysis. Depending on the distribution, either parametric or nonparametric tests were used. The Shapiro-Wilk test can be used to ensure that the differences in pairs from the matched samples are normally distributed.^10^ If this test reveals that the differences in pairs from the matched samples follow a normal distribution, then a paired *t*-test can be used. The Wilcoxon signed-rank test was used to compare differences between each pair of matched samples from a nonparametric perspective.^11^ After the matching process, the number of samples was reduced to 1304 (652 matched cases) because suitable matches could not be found for other cases. In addition, according to the results obtained using the Shapiro-Wilk test, the null hypothesis (H0) was that the test sample was normally distributed. However, these results led us to reject the null hypothesis and accept the alternative hypothesis (H1) that pairs from matched samples did not follow a normal distribution (*P* < .001) in terms of the 4 aspects of OSH. Thus, a nonparametric comparison was suitable for analyzing these matched samples.

## 3. Results

The descriptive statistics for the matched samples are presented in Table 1. Subsequently, a Wilcoxon signed-rank test was conducted, and the results are presented in Table 2. The null hypothesis (H0) of this test was that pairs from the matched sample were equal in terms of the 4 aspects of OSH (H0 = H1). These results showed that the levels of integration of OSH into business management, top management’s leadership and involvement in OSH management, and workers’ participation in OSH management in ISO 45001-certified and non-certified workplaces were all statistically different. The statistical results obtained through this test led us to accept Hypotheses 1–3, as described above. However, the number of injuries and fatalities in ISO 45001-certified and non-certified workplaces was not statistically different. According to the results of the Wilcoxon signed-rank test, there was no statistically significant association between the number of injuries and fatalities and ISO 45001 certification. Therefore, the statistical results failed to support Hypothesis 4. The results did not lead to a rejection of the following claim: ISO 45001-certified and non-certified workplaces did not differ in terms of the number of injuries and fatalities.

## 4. Discussion

This study suggests that although ISO 45001-certified workplaces have a more OSH-friendly internal institutional context than non-certified workplaces, there is no significant difference in the number of injuries and fatalities. These findings can be interpreted in 2 ways.

First, the results show that ISO 45001-certified workplaces do not achieve better OSH performance than non-certified workplaces because ISO 45001 remains in the early stages of expansion, and certification does not require the achievement of OSH performance.^3^ These results may support organizational sociologists’ predictions that meta-standards such as ISO 9001 and ISO 14001 play an symbol to obtain legitimacy from environments.^12^ ISO 45001 has also been adopted symbolically to signal an organization’s responsiveness to societal demand for social responsibility, but not as an instrument for reducing industrial accidents and securing workplace safety and health.^13^^–^^16^ In other words, the results suggest disparities in the organizational context related to workplace safety and health between ISO 45001-certified and non-certified workplaces; they also indicate that there are no significant differences in actual safety and health performance. This implies that ISO 45001 is often used more as a symbol to demonstrate workplace safety rather than as a tool to effectively enhance safety and health performance. However, the results suggest that workplaces that have adopted certification have a more OSH-friendly organizational context. Therefore, ISO 45001-certified workplaces may show a better future OSH performance than non-certified workplaces.

These findings are open to both interpretations. Future research needs to gain a better understanding of the outcomes of ISO 45001. Longitudinal data are needed to explore how ISO 45001 certification and an organization’s internal context for OSH impact OSH performance, such as the number of injuries and fatalities.

### 4.1. Conclusion

Organizations have adopted OSH-related standards, such as OSHA 18001 and ISO 45001, to reduce industrial accidents. However, previous studies reported mixed effects of such standards on industrial accidents. Some studies reported a statistically significant positive effect of OSH certification on reducing industrial accidents, whereas others did not.^17^^–^^19^ Only few studies have investigated the consequences of ISO 45001 because data are not available. Our dataset enabled us to measure the impact of ISO 45001 on multiple aspects and investigate the consequences of ISO 45001. This study contributes to understanding the consequences of ISO 45001, broadly known as the OSH standard.

## Author contributions

I.J. performed all statistical analyses. K.B. developed the theoretical framework and research design.

## Funding

This study was conducted with support from the Ministry of Education of the Republic of Korea and the National Research Foundation of Korea in 2020 (NRF-2020S1A5B8101323)

## Conflicts of interest

The authors report that there are no competing interests to declare.

## Data availability

The data underlying this article will be shared on reasonable request to the corresponding author.
